# Interplay between Trx‐1 and S100P promotes colorectal cancer cell epithelial–mesenchymal transition by up‐regulating S100A4 through AKT activation

**DOI:** 10.1111/jcmm.13541

**Published:** 2018-01-31

**Authors:** Zhigui Zuo, Peili Zhang, Feiyan Lin, Wenjing Shang, Ruichun Bi, Fengying Lu, Jianbo Wu, Lei Jiang

**Affiliations:** ^1^ Department of Colorectal Surgery the First Affiliated Hospital of Wenzhou Medical University Wenzhou China; ^2^ Central Laboratory the First Affiliated Hospital of Wenzhou Medical University Wenzhou China

**Keywords:** colorectal cancer, epithelial–mesenchymal transition, S100A4, S100P, thioredoxin‐1

## Abstract

We previously reported a novel positive feedback loop between thioredoxin‐1 (Trx‐1) and S100P, which promotes the invasion and metastasis of colorectal cancer (CRC). However, the underlying molecular mechanisms remain poorly understood. In this study, we examined the roles of Trx‐1 and S100P in CRC epithelial‐to‐mesenchymal transition (EMT) and their underlying mechanisms. We observed that knockdown of Trx‐1 or S100P in SW620 cells inhibited EMT, whereas overexpression of Trx‐1 or S100P in SW480 cells promoted EMT. Importantly, S100A4 and the phosphorylation of AKT were identified as potential downstream targets of Trx‐1 and S100P in CRC cells. Silencing S100A4 or inhibition of AKT phosphorylation eliminated S100P‐ or Trx‐1‐mediated CRC cell EMT, migration and invasion. Moreover, inhibition of AKT activity reversed S100P‐ or Trx‐1‐induced S100A4 expression. The expression of S100A4 was higher in human CRC tissues compared with their normal counterpart tissues and was significantly correlated with lymph node metastasis and poor survival. The overexpression of S100A4 protein was also positively correlated with S100P or Trx‐1 protein overexpression in our cohort of CRC tissues. In addition, overexpression of S100P reversed the Trx‐1 knockdown‐induced inhibition of S100A4 expression, EMT and migration and invasion in SW620 cells. The data suggest that interplay between Trx‐1 and S100P promoted CRC EMT as well as migration and invasion by up‐regulating S100A4 through AKT activation, thus providing further potential therapeutic targets for suppressing the EMT in metastatic CRC.

## 1 INTRODUCTION

Colorectal cancer (CRC) is one of the most commonly diagnosed cancers worldwide.^1^ Despite the recent advances in diagnosis and treatment, CRC‐related mortality remains high.^2^ Metastasis is one of the major causes of death for CRC patients. Epithelial‐to mesenchymal transition (EMT), a key developmental regulatory programme, has been shown to play an important role in the invasion and metastasis of CRC.^3^ Despite an increased understanding in CRC EMT and metastasis, the underling molecular mechanisms remain poorly understood.

Thioredoxin‐1 (Trx‐1), a 12‐kDa cellular redox protein, is ubiquitously expressed in mammalian cells and has numerous, diverse functions including maintaining cellular redox homeostasis and cell survival.^4^, ^5^ Trx‐1 expression is increased in many tumours where its expression is associated with increased cancer cell growth, inhibited apoptosis and decreased patient survival.^6^, ^7^, ^8^, ^9^, ^10^, ^11^, ^12^ Trx‐1 has been shown to regulate cell growth and survival through the modulation of transcription and cell signalling.^13^, ^14^ We have previously reported a novel positive feedback loop between Trx‐1 and S100P, which promotes CRC invasion and metastasis.^15^ Trx‐1 increases S100P gene transcription through interaction with transcription factor SP1, and S100P in turn promotes the expression and nuclear localization of Trx‐1 through up‐regulation of p‐ERK1/2 and down‐regulation of TXNIP expression.^15^ S100P is a member of the S100 calcium‐binding protein family involved in the regulation of a number of cellular processes.^16^, ^17^, ^18^ S100P expression is up‐regulated in various cancers and has an important role in tumour growth, invasion and metastasis.^19^, ^20^, ^21^ In our previous studies, we found that overexpression of Trx‐1 or S100P enhances CRC cell invasion and metastasis, and conversely, suppression of Trx‐1 or S100P expression inhibits CRC cell invasion and metastasis.^15^, ^19^, ^22^ We also reported that both Trx‐1 and S100P expressions are up‐regulated in CRC and correlates with CRC invasion and metastasis.^15^, ^19^ However, the molecular mechanisms of Trx‐1 and S100P in regulating the invasion and metastasis of CRC need further exploration.

Here, for the first time, we demonstrate that Trx‐1 and S100P promote CRC EMT, migration and invasion by up‐regulating S100A4. This up‐regulation of S100A4 by Trx‐1 and S100P in CRC cells is partly dependent upon AKT activation. Our study reveals a novel mechanism in the Trx‐1/S100P axis that regulates CRC EMT as well as migration and invasion, which may be a potential therapeutic target for the management of CRC.

## 2 MATERIALS AND METHODS

### 2.1 Cell culture and chemicals

Cell lines were purchased from the Type Culture Collection of the Chinese Academy of Sciences, Shanghai, China. The human CRC cell lines SW480 and SW620 are derived from primary (SW480) and metastatic lesions (SW620) of a single colon cancer patient.^23^ Cells were maintained in RPMI 1640 (for SW480 cells) or Dulbecco's modified Eagle's medium (DMEM, for SW620 and HEK293T cells) supplemented with 10% foetal bovine serum (FBS) and 100 U/mL penicillin/streptomycin (Life Technologies, Carlsbad, CA, USA). MK2206 (Cat. # S1078), an AKT inhibitor, was purchased from Selleck Chemicals (Houston, TX, USA).

### 2.2 Lentiviral vector construction and transduction

Lentiviral vector expressing the human Trx‐1 gene or S100P gene, and expressing shRNA sequences targeting Trx‐1 or S100P were cloned as previously described.^15^, ^19^, ^22^ shRNA sequences were targeted to the Trx‐1 mRNA (shTrx‐1: 5′‐GAC TGT CAG GAT GTT GCT TCA GAG TGT GA‐3′)^15^ or S100P mRNA (shS100P: 5′‐AAC TCA CTG AAG TCC ACC TGG GCA TCT CC‐3′).^22^ Lentiviral vectors carrying Enhanced Green Fluorescent Protein (EGFP) gene or shRNA targeting firefly luciferase (shLuc: 5′‐TGC GCT GCT GGT GCC AAC CCT ATT CT‐3′)^22^ were used as controls. Cells were then transduced with the lentivirus as previously described.^15^

### 2.3 Western blotting

For Western blot analysis, cells were lysed on ice using a lysis buffer supplemented with a protease inhibitor cocktail (Sigma‐Aldrich, St. Louis, MO, USA). Total protein (30 μg) was separated by gel electrophoresis and transferred to PVDF membranes. The immunoreaction was carried out using primary antibodies against Trx‐1 (ab133524), S100P (ab133554), S100A4 (ab27957) (Abcam, ITK Diagnostics BV, The Netherlands); AKT (9272), phospho‐AKT (P‐AKT, 4060), β‐Actin (4970) (Cell Signaling Technology, Bioke, The Netherlands); E‐cadherin (610181), vimentin (550513) (BD Biosciences, San Jose, CA, USA); and fibronectin (sc18825) (Santa Cruz Biotechnology, Santa Cruz, CA, USA).

### 2.4 Migration and invasion assay

Cell migration and invasion assays were performed using 24‐well Transwell cell culture chamber inserts with 8 μm pores (Corning Costar Corp., Cambridge, MA, USA) according to the manufacturer's instruction. For the migration assay, 3 × 10^5^ cells per 200 μL in each well were seeded into Transwell inserts and cultured for 24 hours. The bottom chamber of each well was filled with 600 μL of medium containing 10% FBS. Migrated cells were fixed with 4% paraformaldehyde and subsequently stained with 0.1% crystal violet. The invasion assays were carried out using the same methods as the migration assays except that chambers were coated with 1:3 diluted matrigel (BD Biosciences). The migration or invasion cells were counted in 5 random selected fields at a 100 magnification using a microscope. Cell migration and invasion were expressed as relative cell count relative to the each control group. The value from each control group was arbitrarily set as 1. All experiments were performed in triplicates.

### 2.5 siRNA transfection

Cells were transfected with S100A4‐siRNA or control siRNA (negative control, NC) at a final concentration of 50 nM using Lipofectamine 3000 (Invitrogen) according to the manufacturer's protocol. siRNAs were ordered from GenePharma (Shanghai, China). The sequence of siRNA targeting S100A4 was 5′‐AAC GAG GTG GAC TTC CAA GAG‐3′.^24^

### 2.6 Patient recruitment and immunohistochemistry

Immunohistochemistry (IHC) analysis was performed to evaluate the expression of protein in human CRC tissues as described previously.^15^ A total of 112 CRC cases from 1997 to 2003 at the First Affiliated Hospital of Wenzhou Medical University were included. Immunostainings for S100A4, Trx‐1 and S100P were performed on 4 μm sections from paraffin‐embedded tissue samples. The sections were incubated with primary antibodies against S100A4, Trx‐1 or S100P overnight at 4°C. These slides were independently reviewed by 2 trained pathologists and a “H Score”^25^ was used. The H score was obtained by adding the scores of percentage positivity and staining intensity. A cut‐off score was generated by the Receiver Operating Characteristic (ROC) curve analysis, which determined the overexpression of S100A4, Trx‐1 and S100P.^26^ The score with the closest distance from the curve to the point (0.0, 1.0) in the ROC curve which maximizes the sensitivity and specificity as the cut‐off score.^26^ According to ROC curve analysis, the cut‐off scores for Trx‐1, S100P and S100A4 were 118.3, 123.7 and 103.5, respectively.^15^ Recurrence was defined as CRC detected after surgical resection of the patient's primary CRC. Recurrence may be local, lymph nodes or in a distant area. This study was approved by the Ethics Committee of the First Affiliated Hospital of Wenzhou Medical University.

### 2.7 Immunofluorescence staining

Cells grown on coverslips were washed with phosphate‐buffered saline and fixed with 4% paraformaldehyde at 4°C for 20 minutes. Cells were then permeabilized with 0.3% Triton X‐100 and blocked with 10% normal serum. Cells were incubated with primary antibodies to E‐cadherin or vimentin and then incubated with Alexa‐Fluor^®^ 594 goat anti‐rabbit antibody IgG (Life Technologies). Nuclei were counterstained with DAPI (4′,6‐Diamidino‐2‐Phenylindole; Life Technologies). Samples were examined and photographed using a fluorescence microscope (Olympus, Lake Success, NY, USA).

### 2.8 Statistical analysis

All data are presented as mean ± standard deviation. The correlation between protein expression and clinicopathological features was analysed by the chi‐squared test. Comparisons of the continuous variables between 2 groups were performed with an independent samples *t*‐test. One‐way ANOVA followed by post hoc Tukey's or Dunnet's analysis was used for multiple comparisons. A Kaplan‐Meier analysis was used to obtain the survival curve, and the log‐rank test was used for comparing survival curves. A *P* value of less than .05 was considered statistically significant.

## 3 RESULTS

### 3.1 The expression levels of Trx‐1 and S100P influence the EMT phenotype of CRC cells

**Figure 1:**
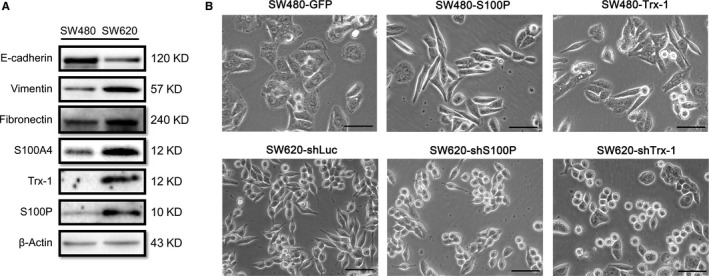
The expression levels of S100P, Trx‐1, S100A4 and EMT‐associated proteins in SW480 and SW620 cells. A, S100P, Trx‐1, S100A4 and EMT‐associated proteins (E‐cadherin, vimentin and fibronectin) were examined by Western blotting. β‐actin was used as the loading control. B, EMT morphological changes induced by S100P or Trx‐1. Representative microscopic views of SW480 and SW620 cells were shown. Scale bar, 50 μm

**Figure 2:**
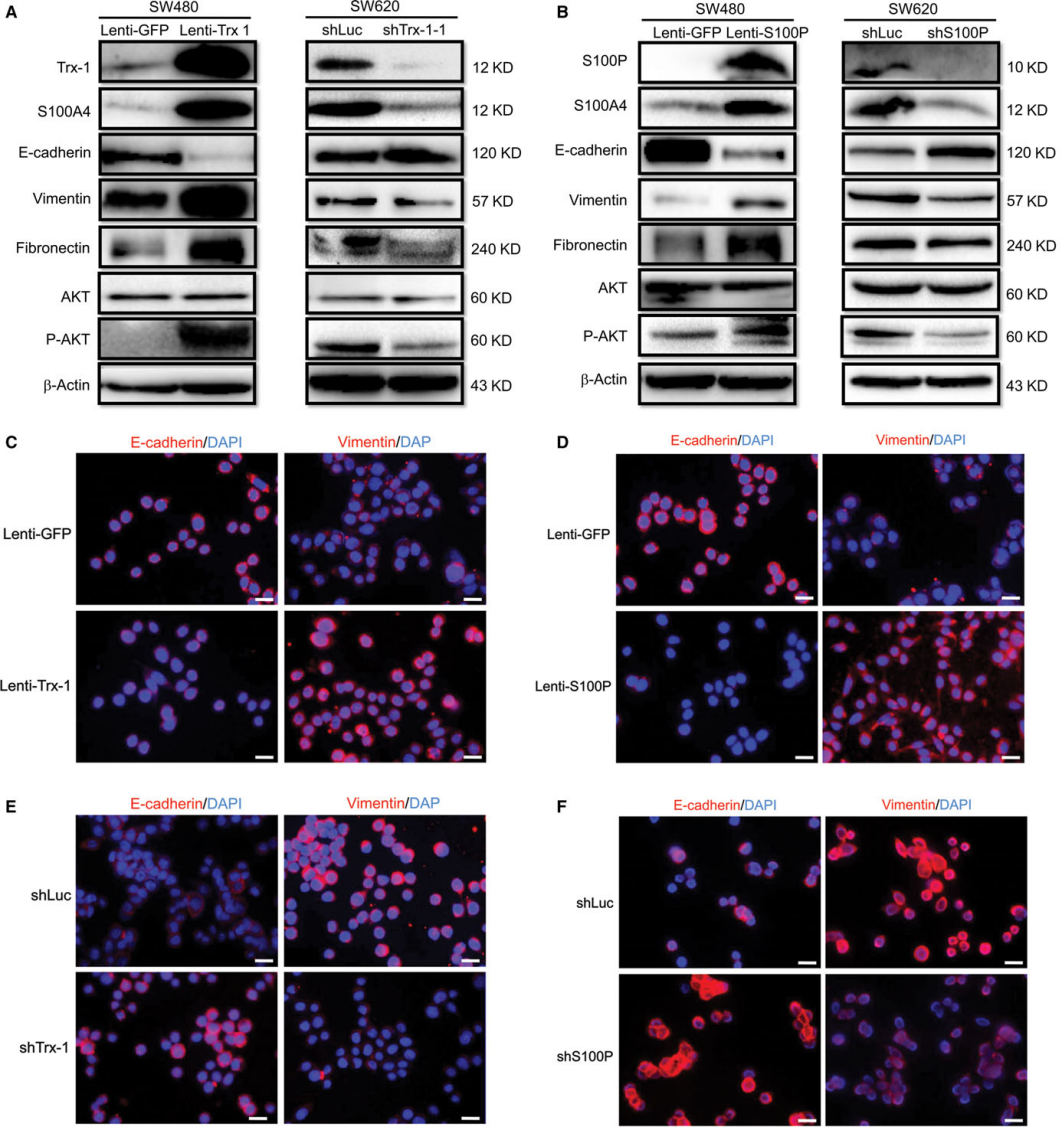
Effects of Trx‐1 and S100P on epithelial–mesenchymal transition of colorectal carcinoma cells. (A) Western blotting revealed that overexpression of Trx‐1 resulted in a decreased expression of epithelial marker E‐cadherin and increased expressions of mesenchymal markers (vimentin and fibronectin), S100A4 and phosphorylated AKT (P‐AKT) in SW480 cells, whereas knockdown of Trx‐1 by shRNA resulted in an increased expression of E‐cadherin and decreased expressions of vimentin, fibronectin, S100A4 and P‐AKT in SW620 cells. (B) Western blotting showed that overexpression of S100P resulted in a decreased expression of E‐cadherin and increased expressions of vimentin, fibronectin, S100A4 and P‐AKT in SW480 cells, whereas knockdown of S100P by shRNA resulted in an increased expression of E‐cadherin and decreased expressions of vimentin, fibronectin, S100A4 and P‐AKT in SW620 cells. β‐Actin was used as the loading control. (C) Immunofluorescence staining of Trx‐1 overexpression down‐regulated E‐cadherin expression while up‐regulating vimentin expression in SW480 cells. (D) Knockdown of Trx‐1 by shRNA up‐regulated E‐cadherin expression and down‐regulated vimentin expression in SW620 cells. (E) S100P overexpression caused a decrease in E‐cadherin expression and an increase in the expression of vimentin in SW480 cells. (F) Knockdown of S100P by shRNA increased the expression of E‐cadherin and decreased vimentin expression in SW620 cells. Nuclei were counterstained using DAPI (blue). Merged figures show the co‐localization of E‐cadherin or vimentin (red) with DAPI. Scale bar = 20 μm

In this study, the CRC cell lines SW480 and SW620 that are derived from primary (SW480) and metastatic lesions (SW620) of the same patient were chosen as model systems for studying EMT.^23^ Protein expression levels were determined by Western‐blot assays, and protein levels relative to β‐actin protein levels were assessed by densitometric analysis. Figure 1A shows that protein levels of S100P, Trx‐1, S100A4, vimentin and fibronectin in the SW620 are higher than that seen in SW480 cells, while the level of epithelial marker E‐cadherin is lower in SW620 than in SW480 cells. As SW480 cells exhibited lower expressions of Trx‐1 and S100P than SW620 cells do, we overexpressed Trx‐1 or S100P in SW480 cells by lentiviral‐mediated gene transfer. Overexpression of S100P or Trx‐1 showed an elongated, mesenchymal morphology as compared to the parental SW480 cells (Figure 1B). In contrast, SW620 cells with S100P or Trx‐1 knockdown showed a reversed EMT morphology: the cells were more epithelial‐like as compared to the control cells (Figure 1B). In addition, ectopic overexpression of Trx‐1 or S100P in SW480 cells resulted in down‐regulation of E‐cadherin, whereas the expressions of the 2 mesenchymal markers vimentin and fibronectin were up‐regulated (Figures 2A and B). On the other hand, knockdown of Trx‐1 or S100P in SW620 by shRNA resulted in an increased expression of E‐cadherin and decreased expressions of vimentin and fibronectin. In addition, overexpression of Trx‐1 or S100P up‐regulated the levels of S100A4 and P‐AKT in SW480 cells, whereas knockdown of Trx‐1 or S100P down‐regulated the levels of S100A4 and P‐AKT in SW620 cells (Figure 2A,B). Moreover, the expression of the mesenchymal marker, vimentin, and the epithelial marker, E‐cadherin, were examined by immunofluorescence. Immunofluorescent staining showed that E‐cadherin expression decreased while vimentin expression increased after the overexpression of Trx‐1 or S100P in SW480 cells (Figure 2C,D). Conversely, knockdown of Trx‐1 or S100P in SW620 cells caused an increase in E‐cadherin expression and a decrease in vimentin expression (Figure 2E,F). These results suggested that S100P or Trx‐1 could induce EMT in CRC cells.

### 3.2 Inhibition of AKT activity caused a mesenchymal‐to‐epithelial transition, and decreased S100A4 expression, and migration and invasion abilities in CRC cells

**Figure 3:**
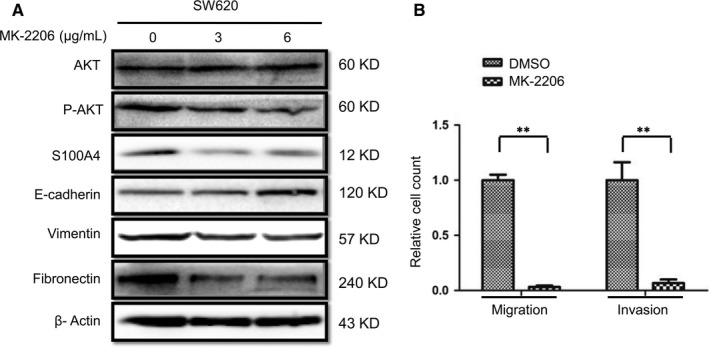
Inhibition of AKT activity induces the mesenchymal‐to‐epithelial transition, decreased S100A4 expression and migration and invasion of SW620 cells. (A) SW620 cells were treated with MK‐2206 (3 μg/mL and 6 μg/mL), an AKT inhibitor, for 24 hours. The expression levels of AKT, phosphorylated AKT (P‐AKT), S100A4, E‐cadherin, vimentin and fibronectin were measured by Western blotting. β‐Actin was used as the loading control. (B) Transwell assays showed that MK‐2206 suppressed SW620 cell migration and invasion. SW620 cells were treated with 3 μg/mL MK‐2206 for 24 hours before performing the Transwell assays (**P ˂ .01). Values represent relative change in migration and invasion normalized to an arbitrary value of 1 for controls

The previous experiments showed that AKT activation and S100A4 expression up‐regulation were observed with Trx‐1‐ or S100P‐induced EMT in CRC cells (Figure 2). These observations lead to the question of whether AKT activation or S100A4 is involved with EMT process and migration and invasion of CRC cells. Figure 3A shows that blocking AKT activity with MK‐2206 in SW620 cells increased the level of E‐cadherin and decreased the levels of the vimentin and fibronectin. In particular, inhibition of AKT activity decreased the expression of S100A4 (Figure 3A) and suppressed SW620 cell migration and invasion (Figure 3B). These results indicate that the AKT activation is associated with the onset of EMT in CRC cells, and S100A4 might be a downstream target of AKT signalling.

### 3.3 Silencing S100A4 by siRNA induces mesenchymal‐to‐epithelial transition and inhibits migration and invasion of SW620 cells

**Figure 4:**
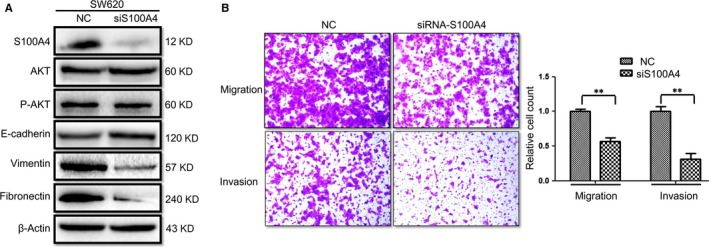
Silencing S100A4 by siRNA induces mesenchymal‐to‐epithelial transition and inhibits migration and invasion of SW620 cells. (A) SW620 cells were transfected with S100A4‐siRNA for 48 hours. The levels of AKT, phosphorylated AKT (P‐AKT), S100A4, E‐cadherin, vimentin and fibronectin were measured by Western blotting. β‐Actin was used as the loading control. (B) Silencing S100A4 by siRNA inhibited migration and invasion rate of SW620 cells by up to 41.2 and 70.5%, respectively. The SW620 cells were transfected with S100A4‐siRNA for 48 hours before performing the Transwell assays (**P ˂ .01)

To study the role of S100A4 in the process of EMT as well as migration and invasion in CRC cells, we used siRNA targeting S100A4 to down‐regulate S100A4 expression. As Figure 4A shows, silencing S100A4 by siRNA in SW620 cells up‐regulated the expression of E‐cadherin but down‐regulated the expressions of vimentin and fibronectin. However, the AKT and P‐AKT protein expressions were not significantly altered (Figure 4A). Transwell assays showed that silencing S100A4 by siRNA inhibited the migration and invasion rate of SW620 cells by up to 41.2 and 70.5%, respectively (Figure 4B).

### 3.4 Clinical and pathological significance of S100A4 expression in CRC

**Figure 5:**
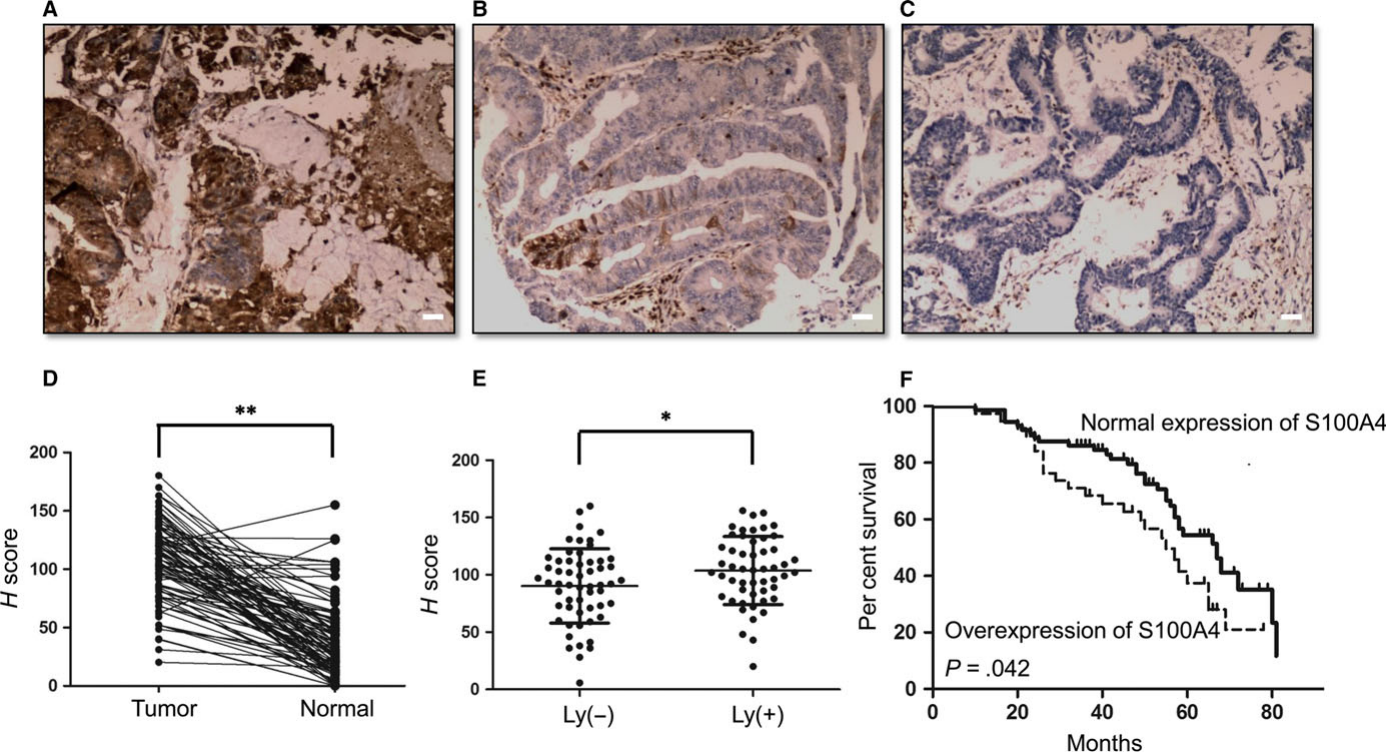
Expression of S100A4 in colorectal carcinoma tissues and its prognostic significance in colorectal cancer patients. (A) Strong immunopositive staining of cancerous tissue, H score = 200; (B) moderate immunopositive staining of cancerous tissue, H score = 120; (C) negative staining of cancerous tissue, H score = 0; D, S100A4 expression of colorectal carcinoma tissue was significantly higher than that of the matched adjacent normal tissues as indicated by IHC. **P < .01. Scale bar = 20 μm. E, Significant up‐regulation of S100A4 protein expression by IHC was shown in colorectal carcinoma with lymph node metastases, relative to colorectal carcinoma without lymph node metastasis. *P < .05. F, Kaplan‐Meier survival analysis according to CRC patients with S100A4 overexpression (log‐rank test, *P* = .042)

To further explore the role of S100A4 in CRC development and progression, S100A4 expression was visualized using IHC for the 112 CRC samples (Figures 5A‐C). We observed that the expression of S100A4 in CRC tissues was significantly higher than that of the matched adjacent normal tissues (Figure 5D, *P* < .01). Significant up‐regulation of S100A4 protein expression was also seen in CRC with lymph node metastases, relative to CRC without lymph node metastasis (Figure 5E, *P* < .05). The elevated expression of S100A4 was significantly correlated with lymph node metastasis (*P* = .045) in CRC (Table 1). Kaplan‐Meier survival analysis showed that an overall survival rate was associated with the S100A4 overexpression patients in comparison with patients with a normal level of S100A4 expression (Figure 5F, *P* = .042). In addition, a significant positive correlation between the overexpression of S100P and S100A4 (Table 2, *P* < .05) or between the overexpression of Trx‐1 and S100A4 (Table 3, *P* < .01) was observed in our cohort of CRC tissues.

**Table 1:** S100A4 expression and clinicopathological parameters in colorectal cancer specimens

	All cases	S100A4 protein		
		Normal expression	Overexpression	*P* value
Sex				
Male	49	33	16	.551
Female	63	39	24	
Histologic grade(WHO)				
Low	96	60	36	.334
High	16	12	4	
Clinical stage				
I–II	73	49	24	.391
III–IV	39	23	16	
pN status				
N0	59	43	16	.045^a^
N1–N2	53	29	24	
Recurrence				
No	58	40	18	.284
Yes	54	32	22	

pN, pathological node.

Statistical analyses were performed by χ^2^ test.

a *P* < .05.

**Table 2:** Correlation between S100P and S100A4 expressions in colorectal cancer tissues

		Cases	S1004		*P* value
			Normal expression	Overexpression	
	Cases		58	33	
S100P	Normal expression	57	41 (70.7%)	16 (48.5%)	˂.05
	Overexpression	34	17 (29.3%)	17 (51.5%)	

**Table 3:** Correlation between Trx‐1 and S100A4 expressions in colorectal cancer tissues

		Cases	S1004		*P* value
			Normal expression	Overexpression	
	Cases		70	40	
Trx‐1	Normal expression	33	28 (40.0%)	5 (12.5%)	˂.01
	Overexpression	77	42 (60.0%)	35 (87.5%)	

### 3.5 Inhibition of AKT activity or silencing S100A4 reverses Trx‐1‐mediated EMT, migration and invasion in CRC cells

**Figure 6:**
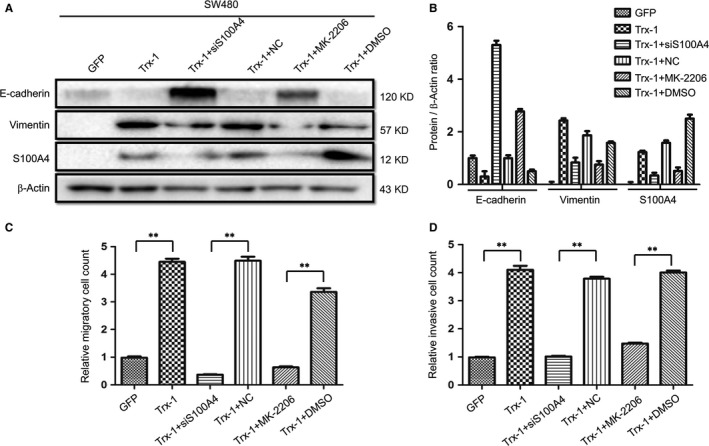
Silencing S100A4 or inhibiting AKT activity reserves Trx‐1‐mediated epithelial–mesenchymal transition (EMT), migration and invasion. A representative Western blot (A) and the summarized data (B) showed that the level of E‐cadherin increased and the levels of the S100A4 and vimentin decreased after silencing S100A4 or blocking AKT phosphorylation with MK‐2206 in SW480‐Trx‐1 cells. SW480‐Trx‐1 cells were transfected with 50 μM S100A4‐siRNA for 48 hours or treated with AKT inhibitor MK‐2206 (6 μg/mL). β‐Actin was used as the loading control. (C,D) Transwell assays demonstrated that the enhanced migration and invasive abilities of SW480‐Trx‐1 cells were inhibited after silencing S100A4 or blocking the phosphorylation of AKT

We further examined whether Trx‐1 and S100P promote CRC cell EMT, migration and invasion via up‐regulating S100A4 expression and activating AKT. We then silenced S100A4 using siRNA targeting S100A4 or blocked the phosphorylation of AKT with an AKT inhibitor MK‐2206 in SW480‐Trx‐1 and SW480‐S100P cells. Following siRNA‐S100A4 or MK‐2206 treatment, Trx‐1‐induced EMT was reversed, as evidenced by the increased expression of the epithelial marker, E‐cadherin and the decreased expression of the mesenchymal marker, vimentin, in SW480‐Trx‐1 cells (Figure 6A,B). Moreover, blocking AKT phosphorylation with MK‐2206 reversed Trx‐1‐induced S100A4 expression. In addition, Transwell assays showed that silencing S100A4 or blocking the phosphorylation of AKT significantly impaired Trx‐1‐induced migration and invasion of SW480 cells (Figure 6C,D).

### 3.6 Inhibition of AKT activity or silencing S100A4 reverses S100P‐mediated EMT, migration and invasion in CRC cells

**Figure 7:**
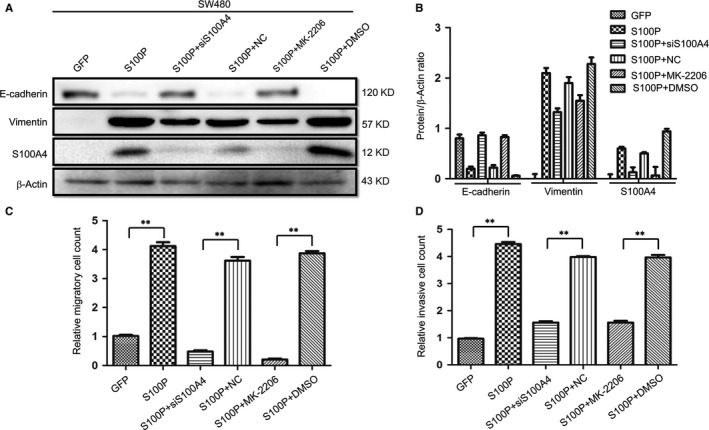
Silencing S100A4 or inhibiting AKT activity reverses S100P‐mediated EMT, migration and invasion. A representative Western blot (A) and the summarized data (B) showed that the level of E‐cadherin increased and the levels of S100A4 and vimentin decreased after silencing S100A4 or blocking phosphorylation of AKT with MK‐2206 in SW480‐S100P cells. SW480‐S100P cells were transfected with 50 μM S100A4‐siRNA for 48 hours or treated with AKT inhibitor MK‐2206 (6 μg/mL). β‐Actin was used as the loading control. (C, D) Transwell assays showed that the enhanced migration and invasive abilities of SW480‐S100P cells were inhibited after silencing S100A4 or blocking phosphorylation of AKT

After siRNA‐S100A4 or MK‐2206 treatment, S100P‐induced EMT was also reversed in SW480‐S100P cells (Figure 7A,B). Moreover, inhibition of AKT activity reversed S100P‐induced S100A4 expression. Transwell assays showed that silencing S100A4 or inhibition of AKT activity significantly suppressed the S100P‐induced migration and invasion of SW480 cells (Figure 7C,D).

### 3.7 Ectopic expression of S100P partially reverses the inhibition of EMT, migration and invasion caused by Trx‐1 knockdown

**Figure 8:**
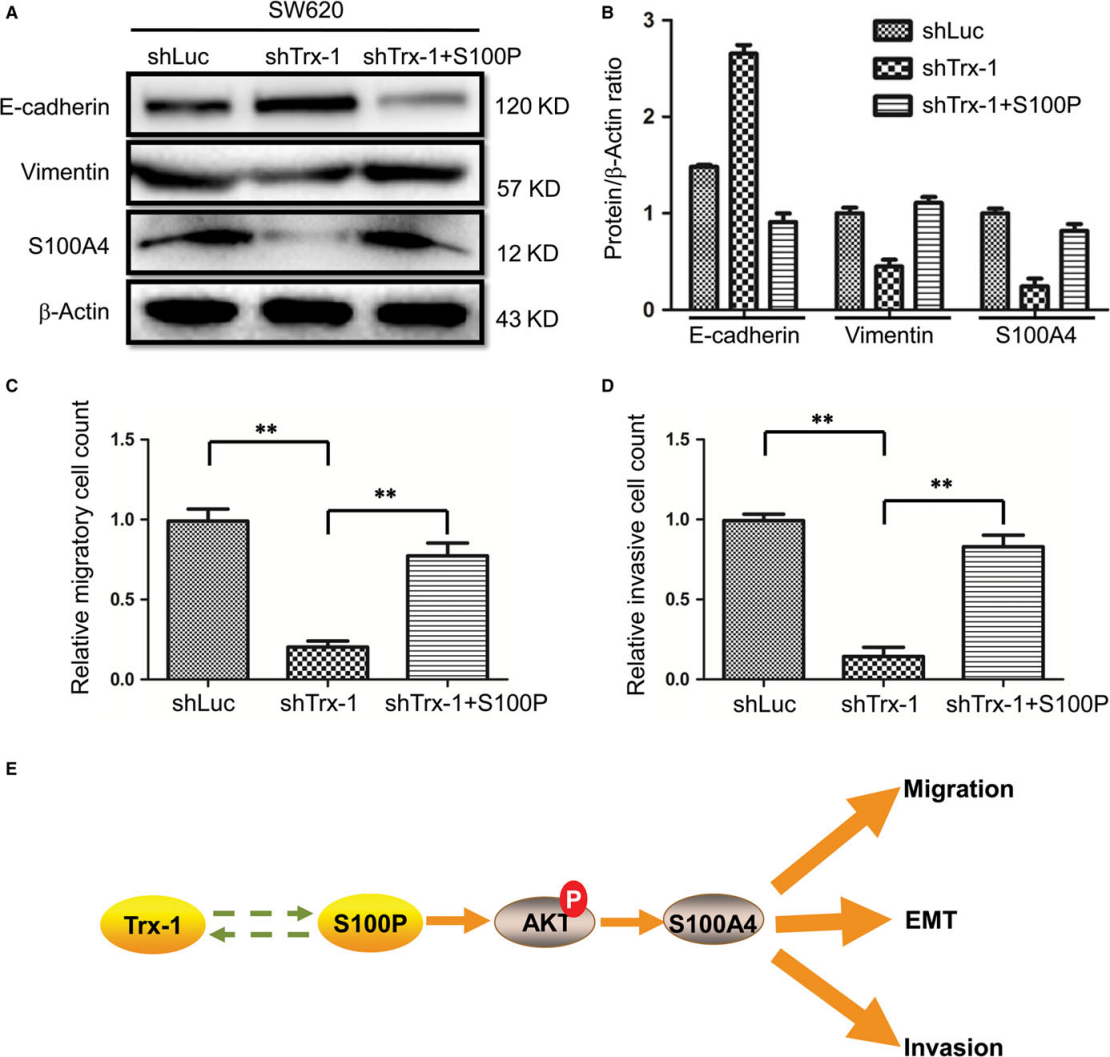
S100P partially reverses the inhibition of EMT, migration and invasion caused by Trx‐1 knockdown. A representative Western blot (A) and the summarized data (B) showed that overexpression of S100P partially reversed Trx‐1 silencing‐induced mesenchymal–epithelial transition in SW620 cells. SW620‐shTrx‐1 cells were transfected with Lenti‐S100P; the expression levels of E‐cadherin, S100A4 and vimentin were assessed by Western blotting. β‐Actin was used as the loading control. (C, D) Overexpression of S100P partly reversed Trx‐1 silencing‐induced inhibition of migration and invasion in SW620 cells. (E) Model for crosstalk between S100P and Trx‐1 that ultimately promotes colorectal cancer cell EMT, migration and invasion by up‐regulating S100A4 through AKT activation

When S100P was introduced into Trx‐1‐silenced SW620 cells, the inhibited EMT of CRC cells and S100A4 expression was substantially re‐enhanced (Figure 8A,B). Also, ectopic expression of S100P partially reversed Trx‐1 knockdown‐induced inhibition of migration and invasion in SW620 cells (Figure 8C,D).

## 4 DISCUSSION

Epithelial‐to‐mesenchymal transition, an intricate process by which epithelial cells lose epithelial characteristics and gain a migratory mesenchymal phenotype, plays a key role in cancer invasion and metastasis.^27^, ^28^, ^29^ EMT is characterized by a decreased expression of epithelial markers, for example E‐cadherin, and increased expressions of mesenchymal markers, for example vimentin and fibronectin.^30^ Preventing or reversing EMT processes might be a promising therapeutic strategy for metastatic CRC treatment.^3^ Trx‐1 is an essential component of the Trx system which plays crucial roles in the regulation of cellular redox signalling pathways.^31^ Trx1 is mainly located in cytosol, and it is involved in maintaining redox homoeostasis and interacts with several proteins related to oxidative stress, cell proliferation and apoptosis, such as thioredoxin‐interacting protein (TXNIP),^32^ apoptosis signal‐regulated kinase (ASK‐1),^33^ SENP1 34 and PTEN^35^ thereby regulating their functions. In response to oxidative stress, Trx1 is translocated into the nucleus and activates several transcription factors, such as NF‐κB, p53, AP‐1, glucocorticoid receptor and SP1, by promoting their DNA binding activity.^36^, ^37^, ^38^

Our previous report showed a positive feedback mechanism between Trx‐1 and S100P, which promotes CRC invasion and metastasis.^15^ Ectopic expression of Trx‐1 promotes CRC cell migration and invasions in vitro and liver metastasis in vivo. Conversely, Trx‐1 knockdown inhibits CRC cell migration and invasion abilities in vitro and liver metastasis in vivo.^15^ Similar results were also obtained in CRC cells with S100P overexpression or knockdown.^19^, ^22^ Trx‐1 promotes CRC invasion and metastasis through crosstalk with S100P.^15^ These results suggested that interplay between Trx‐1 and S100P plays important roles in CRC metastasis. It has been reported that S100P acts as a ligand for the receptor for advanced glycation endproducts (RAGE) and activates ERK1/2, NF‐kB and the JAK/STAT pathway.^39^, ^40^, ^41^ The S100P/RAGE signalling pathway contributes to cancer progression by triggering the oncogenic miR‐155^42^ and miR‐21^43^ in colon cancer. S100P has also been reported to interact with integrin α7,^21^ myosin II^44^ or EZRIN,^45^ leading to increase cell migration. Here, we report yet another novel mechanism wherein Trx‐1 and S100P promote CRC EMT as well as migration and invasion by up‐regulating S100A4 through the activation of AKT.

SW480 and SW620 colon carcinoma cell lines are derived from primary and secondary tumours resected from the same patient.^23^ SW480 cells are mainly epithelial, whereas SW620 cells are mesenchymal.^46^, ^47^ The increase in the expression levels of Trx‐1, S100P and S100A4 were observed in SW620 cells compared with SW480 cells. Therefore, we overexpressed Trx‐1 or S100P in SW480 cells and silenced Trx‐1 or S100P in SW620 cells. Our results showed that down‐regulation of Trx‐1 or S100P in SW620 cells inhibited EMT, whereas overexpression of Trx‐1 or S100P in SW480 cells promoted EMT. S100P was reported to promote EMT and the invasion and metastasis of CRC by activating RAGE/ERK signalling.^48^ Moreover, Jiang et al. demonstrated that Trx‐1 is a critical mediator of TGF‐beta induced EMT in salivary adenoid cystic carcinoma.^49^ S100P could promote cancer invasion and metastasis by promoting the EMT in CRC^48^, ^50^ and lung cancer.^21^

In this study, we saw that the down‐regulation of S100P or Trx‐1 inhibited S100A4 expression, whereas overexpression of S100P or Trx‐1 promoted S100A4 expression. Down‐regulation of S100A4 expression by siRNA reversed S100P‐ and Trx‐1‐induced EMT, migration and invasion of CRC cells. S100A4 is also a ubiquitous small, calcium‐binding protein that is directly involved in tumour metastasis via increased cell motility and invasion.^51^ S100A4 is regarded as an important regulator of metastasis and EMT.^51^, ^52^, ^53^ Overexpression of S100A4 promotes metastasis in several experimental animal models, in contrast, down‐regulation of S100A4 expression reduces the metastatic capacity of cancer cells.^24^, ^51^, ^54^, ^55^, ^56^, ^57^, ^58^ In CRC, S100A4 is overexpressed and its expression is correlated with patient outcome.^59^, ^60^ Consistent with that, our results showed that the expression of S100A4 was higher in human CRC tissues compared with their normal counterpart tissues, and higher expression of S100A4 was significantly correlated with lymph node metastasis and poor survival. Furthermore, the overexpression of S100A4 protein was positively correlated with S100P or Trx‐1 protein overexpression in our cohort of CRC tissues. Dahlmann M et al. have reported that stable knockdown of S100A4 in CRC HCT116 cells by transfection with S100A4‐shRNA expression plasmids resulted in reduced the liver metastatic potential of CRC cells when intrasplenically transplanted in mice.^61^ Moreover, hydrodynamics‐based systemic treatment with plasmids DNA for S100A4‐specific shRNA, via repeated tail vein injection, inhibited the formation of liver metastases.^61^ These results suggest that S100A4 may be a critical downstream target of S100P and Trx‐1 and is responsible for the S100P‐ or Trx‐1‐induced EMT and invasiveness of CRC cells.

Next, we tried to elucidate the underlying mechanism by which Trx‐1 and S100P regulates S100A4. Our results further showed that S100P or Trx‐1 could regulate AKT phosphorylation, and blocking AKT signalling with MK‐2206 down‐regulated S100A4 expression and inhibited EMT as well as migration and invasion of SW620 cells. Liu S et al. reported that HBXIP induces S100A4 expression through PI3K/AKT signalling pathway in breast cancer cells.^62^ S100P could also activate AKT signalling in lung cancer cells.^21^ Indeed, we observed that blocking AKT signalling partially reversed S100P‐ or Trx‐1‐induced S100A4 expression, EMT, and migration and invasion of CRC cells. Therefore, we speculate that AKT signalling may be involved in S100A4 up‐regulation and EMT mediated by S100P or Trx‐1. Further, we demonstrated that overexpression of S100P could reverse the Trx‐1 knockdown‐induced inhibition of S100A4 expression, EMT, and migration and invasion of CRC cells.

In summary, our findings demonstrate for the first time a novel molecular mechanism involving Trx‐1 and S100P in the regulation of the invasion and metastasis capabilities of CRC cells. EMT has been shown to play a critical role in tumour metastasis, and inhibition of EMT is considered to be a promising approach to prevent metastasis.^63^ The identification of functionally relevant regulators of EMT may offer new appropriate targets and therapeutic opportunities for controlling cancer progression and metastasis.^63^ Our results demonstrate that the interplay between S100P and Trx‐1 promotes CRC cell EMT, migration and invasion by up‐regulating S100A4 through AKT activation (Figure 8E), suggesting that the inhibition of AKT or S100A4 may serve as important therapeutic strategies in suppressing the EMT in metastatic CRC with S100P or Trx‐1 overexpression.

## CONFLICT OF INTEREST

The authors confirm that there are no conflicts of interest.

## References

[1] Haggar FA , Boushey RP . Colorectal cancer epidemiology: incidence, mortality, survival, and risk factors. Clin Colon Rectal Surg. 2009;22:191‐197. 21037809 10.1055/s-0029-1242458 PMC2796096

[2] Khair G , Monson JR , Greenman J . Epithelial molecular markers in the peripheral blood of patients with colorectal cancer. Dis Colon Rectum. 2007;50:1188‐1203. 17436048 10.1007/s10350-006-0875-9

[3] Cao H , Xu E , Liu H , et al. Epithelial‐mesenchymal transition in colorectal cancer metastasis: a system review. Pathol Res Pract. 2015;211:557‐569. 26092594 10.1016/j.prp.2015.05.010

[4] Zhao L , Li W , Zhou Y , et al. The overexpression and nuclear translocation of Trx‐1 during hypoxia confers on HepG2 cells resistance to DDP, and GL‐V9 reverses the resistance by suppressing the Trx‐1/Ref‐1 axis. Free Radic Biol Med. 2015;82:29‐41. 25656992 10.1016/j.freeradbiomed.2015.01.014

[5] Li C , Thompson MA , Tamayo AT , et al. Over‐expression of Thioredoxin‐1 mediates growth, survival, and chemoresistance and is a druggable target in diffuse large B‐cell lymphoma. Oncotarget. 2012;3:314‐326. 22447839 10.18632/oncotarget.463 PMC3359887

[6] Watanabe R , Nakamura H , Masutani H , et al. Anti‐oxidative, anti‐cancer and anti‐inflammatory actions by thioredoxin 1 and thioredoxin‐binding protein‐2. Pharmacol Ther. 2010;127:261‐270. 20435060 10.1016/j.pharmthera.2010.04.004

[7] Noike T , Miwa S , Soeda J , et al. Increased expression of thioredoxin‐1, vascular endothelial growth factor, and redox factor‐1 is associated with poor prognosis in patients with liver metastasis from colorectal cancer. Hum Pathol. 2008;39:201‐208. 17949784 10.1016/j.humpath.2007.04.024

[8] Kim HJ , Chae HZ , Kim YJ , et al. Preferential elevation of Prx I and Trx expression in lung cancer cells following hypoxia and in human lung cancer tissues. Cell Biol Toxicol. 2003;19:285‐298. 14703116 10.1023/b:cbto.0000004952.07979.3d

[9] Han H , Bearss DJ , Browne LW , et al. Identification of differentially expressed genes in pancreatic cancer cells using cDNA microarray. Can Res. 2002;62:2890‐2896. 12019169

[10] Raffel J , Bhattacharyya AK , Gallegos A , et al. Increased expression of thioredoxin‐1 in human colorectal cancer is associated with decreased patient survival. J Lab Clin Med. 2003;142:46‐51. 12878985 10.1016/S0022-2143(03)00068-4

[11] Cha MK , Suh KH , Kim IH . Overexpression of peroxiredoxin I and thioredoxin1 in human breast carcinoma. J Exp Clin Cancer Res. 2009;28:93. 19566940 10.1186/1756-9966-28-93 PMC2711968

[12] Grogan TM , Fenoglio‐Prieser C , Zeheb R , et al. Thioredoxin, a putative oncogene product, is overexpressed in gastric carcinoma and associated with increased proliferation and increased cell survival. Hum Pathol. 2000;31:475‐481. 10821495 10.1053/hp.2000.6546

[13] Welsh SJ , Bellamy WT , Briehl MM , et al. The redox protein thioredoxin‐1 (Trx‐1) increases hypoxia‐inducible factor 1alpha protein expression: Trx‐1 overexpression results in increased vascular endothelial growth factor production and enhanced tumor angiogenesis. Can Res. 2002;62:5089‐5095. 12208766

[14] Powis G , Kirkpatrick DL . Thioredoxin signaling as a target for cancer therapy. Curr Opin Pharmacol. 2007;7:392‐397. 17611157 10.1016/j.coph.2007.04.003

[15] Lin F , Zhang P , Zuo Z , et al. Thioredoxin‐1 promotes colorectal cancer invasion and metastasis through crosstalk with S100P. Cancer Lett. 2017;401:1‐10. 28483515 10.1016/j.canlet.2017.04.036

[16] Jiang H , Hu H , Tong X , et al. Calcium‐binding protein S100P and cancer: mechanisms and clinical relevance. J Cancer Res Clin Oncol. 2012;138:1‐9. 21947242 10.1007/s00432-011-1062-5 PMC11824467

[17] Wu Z , Boonmars T , Nagano I , et al. Significance of S100P as a biomarker in diagnosis, prognosis and therapy of opisthorchiasis‐associated cholangiocarcinoma. Int J Cancer. 2016;138:396‐408. 26312563 10.1002/ijc.29721

[18] Prica F , Radon T , Cheng Y , et al. The life and works of S100P – from conception to cancer. Am J Cancer Res. 2016;6:562‐576. 27186425 PMC4859681

[19] Dong L , Wang F , Yin X , et al. Overexpression of S100P promotes colorectal cancer metastasis and decreases chemosensitivity to 5‐FU in vitro. Mol Cell Biochem. 2014;389:257‐264. 24381058 10.1007/s11010-013-1947-5

[20] Barry S , Chelala C , Lines K , et al. S100P is a metastasis‐associated gene that facilitates transendothelial migration of pancreatic cancer cells. Clin Exp Metastasis. 2013;30:251‐264. 23007696 10.1007/s10585-012-9532-y

[21] Hsu YL , Hung JY , Liang YY , et al. S100P interacts with integrin alpha7 and increases cancer cell migration and invasion in lung cancer. Oncotarget. 2015;6:29585‐29598. 26320193 10.18632/oncotarget.4987 PMC4745748

[22] Jiang L , Lai YK , Zhang J , et al. Targeting S100P inhibits colon cancer growth and metastasis by Lentivirus‐mediated RNA interference and proteomic analysis. Mol Med. 2011;17:709‐716. 21327297 10.2119/molmed.2011.00008 PMC3146612

[23] Hewitt RE , McMarlin A , Kleiner D , et al. Validation of a model of colon cancer progression. J Pathol. 2000;192:446‐454. 11113861 10.1002/1096-9896(2000)9999:9999<::AID-PATH775>3.0.CO;2-K

[24] Saleem M , Kweon MH , Johnson JJ , et al. S100A4 accelerates tumorigenesis and invasion of human prostate cancer through the transcriptional regulation of matrix metalloproteinase 9. Proc Natl Acad Sci USA. 2006;103:14825‐14830. 16990429 10.1073/pnas.0606747103 PMC1595436

[25] Su EJ , Ernst L , Abdallah N , et al. Estrogen receptor‐beta and fetoplacental endothelial prostanoid biosynthesis: a link to clinically demonstrated fetal growth restriction. J Clin Endocrinol Metab. 2011;96:E1558‐E1567. 21832119 10.1210/jc.2011-1084 PMC3200254

[26] Zlobec I , Steele R , Terracciano L , Jass JR , Lugli A . Selecting immunohistochemical cut‐off scores for novel biomarkers of progression and survival in colorectal cancer. J Clin Pathol. 2007;60:1112‐1116. 17182662 10.1136/jcp.2006.044537 PMC2014838

[27] Gupta GP , Massague J . Cancer metastasis: building a framework. Cell. 2006;127:679‐695. 17110329 10.1016/j.cell.2006.11.001

[28] Thiery JP , Acloque H , Huang RY , et al. Epithelial‐mesenchymal transitions in development and disease. Cell. 2009;139:871‐890. 19945376 10.1016/j.cell.2009.11.007

[29] Smith BN , Bhowmick NA . Role of EMT in metastasis and therapy resistance. J Clin Med. 2016;5:17. 10.3390/jcm5020017 PMC4773773 26828526

[30] Lamouille S , Xu J , Derynck R . Molecular mechanisms of epithelial‐mesenchymal transition. Nat Rev Mol Cell Biol. 2014;15:178‐196. 24556840 10.1038/nrm3758 PMC4240281

[31] Zhang J , Li X , Han X , et al. Targeting the thioredoxin system for cancer therapy. Trends Pharmacol Sci. 2017;38:794‐808. 28648527 10.1016/j.tips.2017.06.001

[32] Nishiyama A , Matsui M , Iwata S , et al. Identification of thioredoxin‐binding protein‐2/vitamin D(3) up‐regulated protein 1 as a negative regulator of thioredoxin function and expression. J Biol Chem. 1999;274:21645‐21650. 10419473 10.1074/jbc.274.31.21645

[33] Saitoh M , Nishitoh H , Fujii M , et al. Mammalian thioredoxin is a direct inhibitor of apoptosis signal‐regulating kinase (ASK) 1. EMBO J. 1998;17:2596‐2606. 9564042 10.1093/emboj/17.9.2596 PMC1170601

[34] Li X , Luo Y , Yu L , et al. SENP1 mediates TNF‐induced desumoylation and cytoplasmic translocation of HIPK1 to enhance ASK1‐dependent apoptosis. Cell Death Differ. 2008;15:739‐750. 18219322 10.1038/sj.cdd.4402303

[35] Meuillet EJ , Mahadevan D , Berggren M , et al. Thioredoxin‐1 binds to the C2 domain of PTEN inhibiting PTEN's lipid phosphatase activity and membrane binding: a mechanism for the functional loss of PTEN's tumor suppressor activity. Arch Biochem Biophys. 2004;429:123‐133. 15313215 10.1016/j.abb.2004.04.020

[36] Schroeder P , Popp R , Wiegand B , et al. Nuclear redox‐signaling is essential for apoptosis inhibition in endothelial cells–important role for nuclear thioredoxin‐1. Arterioscler Thromb Vasc Biol. 2007;27:2325‐2331. 17823364 10.1161/ATVBAHA.107.149419

[37] Zschauer TC , Matsushima S , Altschmied J , et al. Interacting with thioredoxin‐1–disease or no disease? Antioxid Redox Signal. 2013;18:1053‐1062. 22867430 10.1089/ars.2012.4822 PMC3567779

[38] Farina AR , Cappabianca L , DeSantis G , et al. Thioredoxin stimulates MMP‐9 expression, de‐regulates the MMP‐9/TIMP‐1 equilibrium and promotes MMP‐9 dependent invasion in human MDA‐MB‐231 breast cancer cells. FEBS Lett. 2011;585:3328‐3336. 21963718 10.1016/j.febslet.2011.09.023

[39] Fuentes MK , Nigavekar SS , Arumugam T , et al. RAGE activation by S100P in colon cancer stimulates growth, migration, and cell signaling pathways. Dis Colon Rectum. 2007;50:1230‐1240. 17587138 10.1007/s10350-006-0850-5

[40] Arumugam T , Simeone DM , Schmidt AM , et al. S100P stimulates cell proliferation and survival via receptor for activated glycation end products (RAGE). J Biol Chem. 2004;279:5059‐5065. 14617629 10.1074/jbc.M310124200

[41] Arumugam T , Ramachandran V , Gomez SB , et al. S100P‐derived RAGE antagonistic peptide reduces tumor growth and metastasis. Clin Cancer Res. 2012;18:4356‐4364. 22718861 10.1158/1078-0432.CCR-12-0221 PMC3845828

[42] Onyeagucha BC , Mercado‐Pimentel ME , Hutchison J , et al. S100P/RAGE signaling regulates microRNA‐155 expression via AP‐1 activation in colon cancer. Exp Cell Res. 2013;319:2081‐2090. 23693020 10.1016/j.yexcr.2013.05.009 PMC3726211

[43] Mercado‐Pimentel ME , Onyeagucha BC , Li Q , et al. The S100P/RAGE signaling pathway regulates expression of microRNA‐21 in colon cancer cells. FEBS Lett. 2015;589:2388‐2393. 26193421 10.1016/j.febslet.2015.07.010 PMC4582666

[44] Du M , Wang G , Ismail TM , et al. S100P dissociates myosin IIA filaments and focal adhesion sites to reduce cell adhesion and enhance cell migration. J Biol Chem. 2012;287:15330‐15344. 22399300 10.1074/jbc.M112.349787 PMC3346139

[45] Austermann J , Nazmi AR , Muller‐Tidow C , et al. Characterization of the Ca2+ ‐regulated ezrin‐S100P interaction and its role in tumor cell migration. J Biol Chem. 2008;283:29331‐29340. 18725408 10.1074/jbc.M806145200 PMC2662020

[46] Kubens BS , Zanker KS . Differences in the migration capacity of primary human colon carcinoma cells (SW480) and their lymph node metastatic derivatives (SW620). Cancer Lett. 1998;131:55‐64. 9839620 10.1016/s0304-3835(98)00201-8

[47] Roy N , Bommi PV , Bhat UG , et al. DDB2 suppresses epithelial‐to‐mesenchymal transition in colon cancer. Can Res. 2013;73:3771‐3782. 10.1158/0008-5472.CAN-12-4069 PMC3686976 23610444

[48] Shen ZY , Fang Y , Zhen L , et al. Analysis of the predictive efficiency of S100P on adverse prognosis and the pathogenesis of S100P‐mediated invasion and metastasis of colon adenocarcinoma. Cancer Genet. 2016;209:143‐153. 26975699 10.1016/j.cancergen.2016.02.002

[49] Jiang Y , Feng X , Zheng L , et al. Thioredoxin 1 mediates TGF‐beta‐induced epithelial‐mesenchymal transition in salivary adenoid cystic carcinoma. Oncotarget. 2015;6:25506‐25519. 26325518 10.18632/oncotarget.4635 PMC4694848

[50] Shen Z , Deng H , Fang Y , et al. Identification of the interplay between SOX9 and S100P in the metastasis and invasion of colon carcinoma. Oncotarget. 2015;6:20672‐20684. 26009899 10.18632/oncotarget.3967 PMC4653034

[51] Boye K , Maelandsmo GM . S100A4 and metastasis: a small actor playing many roles. Am J Pathol. 2010;176:528‐535. 20019188 10.2353/ajpath.2010.090526 PMC2808059

[52] Hua T , Liu S , Xin X , et al. S100A4 promotes endometrial cancer progress through epithelial‐mesenchymal transition regulation. Oncol Rep. 2016;35:3419‐3426. 27109209 10.3892/or.2016.4760

[53] Xu H , Li M , Zhou Y , et al. S100A4 participates in epithelial‐mesenchymal transition in breast cancer via targeting MMP2. Tumour Biol. 2016;37:2925‐2932. 26409452 10.1007/s13277-015-3709-3

[54] Lo JF , Yu CC , Chiou SH , et al. The epithelial‐mesenchymal transition mediator S100A4 maintains cancer‐initiating cells in head and neck cancers. Can Res. 2011;71:1912‐1923. 10.1158/0008-5472.CAN-10-2350 21169409

[55] Yu LJ , Li Y , Li C , et al. Restoration of S100A4 expression enhances invasive and metastatic potentials of COLO16 cutaneous squamous cancer cells. Cancer Biomark. 2014;14:325‐333. 25171474 10.3233/CBM-140414 PMC12928329

[56] Che P , Yang Y , Han X , et al. S100A4 promotes pancreatic cancer progression through a dual signaling pathway mediated by Src and focal adhesion kinase. Sci Rep. 2015;5:8453. 25677816 10.1038/srep08453 PMC4326725

[57] Orre LM , Panizza E , Kaminskyy VO , et al. S100A4 interacts with p53 in the nucleus and promotes p53 degradation. Oncogene. 2013;32:5531‐5540. 23752197 10.1038/onc.2013.213

[58] Zhang K , Yu M , Hao F , et al. Knockdown of S100A4 blocks growth and metastasis of anaplastic thyroid cancer cells in vitro and in vivo. Cancer Biomark. 2016;17:281‐291. 27802204 10.3233/CBM-160640 PMC13020503

[59] Stein U , Arlt F , Walther W , et al. The metastasis‐associated gene S100A4 is a novel target of beta‐catenin/T‐cell factor signaling in colon cancer. Gastroenterology. 2006;131:1486‐1500. 17101323 10.1053/j.gastro.2006.08.041

[60] Gongoll S , Peters G , Mengel M , et al. Prognostic significance of calcium‐binding protein S100A4 in colorectal cancer. Gastroenterology. 2002;123:1478‐1484. 12404222 10.1053/gast.2002.36606

[61] Dahlmann M , Sack U , Herrmann P , et al. Systemic shRNA mediated knock down of S100A4 in colorectal cancer xenografted mice reduces metastasis formation. Oncotarget. 2012;3:783‐797. 22878175 10.18632/oncotarget.572 PMC3478456

[62] Liu S , Li L , Zhang Y , et al. The oncoprotein HBXIP uses two pathways to up‐regulate S100A4 in promotion of growth and migration of breast cancer cells. J Biol Chem. 2012;287:30228‐30239. 22740693 10.1074/jbc.M112.343947 PMC3436276

[63] Tsai JH , Yang J . Epithelial‐mesenchymal plasticity in carcinoma metastasis. Genes Dev. 2013;27:2192‐2206. 24142872 10.1101/gad.225334.113 PMC3814640

